# Routine Cysticotomy and Flushing of the Cystic Duct in Patients with Low Risk of Common Duct Stones: Can It Be Beneficial?

**DOI:** 10.1155/2017/9814389

**Published:** 2017-07-11

**Authors:** Piera Leon, Fabiola Giudici, Antonio Sciuto, Francesco Corcione

**Affiliations:** ^1^Department of Medical, Surgical and Health Sciences, General Surgery Clinic, University of Trieste, Trieste, Italy; ^2^Department of General Surgery, “Azienda Ospedaliera dei Colli”, Monaldi Hospital, Naples, Italy

## Abstract

**Background:**

Gallstone disease affects 15–20% of the general population and up to 20% of these patients present common bile duct stones.

**Aim:**

This observational study reports our experience on routine cysticotomy and flushing of the cystic duct in patients with low risk of common duct stones.

**Materials and Methods:**

We analyzed 731 patients who underwent laparoscopic cholecystectomy between September 2013 and September 2015.

**Results:**

Patients were preoperatively stratified on the clinical risk; those presenting with low preoperative risk of common bile duct stones were referred to undergo laparoscopic cholecystectomy and routine cysticotomy with bile duct flushing. Patients presenting thick bile sludge, solid debrides, and/or increased tension of bile outflow underwent unplanned cholangiography. No intraoperative complications or conversion to open technique occurred. Average follow-up time was 22,8 months (range 12 to 37). Rate of retained ductal stones accounted for 0,3%.

**Conclusions:**

Routine cysticotomy and bile flushing in our experience is a valid, simple, and not time consuming manoeuvre that can help decompressing and flushing CBD. Moreover, it is a valid tool for extending selective IOC approach in a focused manner. Further evaluations have to be conducted to evaluate risks and effectiveness of this manoeuvre.

## 1. Introduction

Gallstone disease (GSD) is one of the most common biliary tract disorders affecting 15–20% of the general population in both Western and Eastern Countries.

Moreover, up to 20% of these patients present simultaneously common bile duct stones (CBDS) which can lead to many severe life-threatening conditions, such as acute pancreatitis, jaundice, ascending cholangitis, and hepatic abscesses [1, 2].

In the era of laparoscopic treatment for cholelithiasis (more than 80% of the cholecystectomies are laparoscopic in Western Countries), intraoperative cholangiography (IOC) and laparoscopic common bile duct exploration (LCBDE) have been advocated to diagnose and manage CBDS in a single stage [3]: this strategy shows excellent outcomes in CBD clearance, morbidity, and mortality. The single stage strategy permits shorter hospital stay and fewer procedures and is more cost-effective in comparison to the two-stage strategy provided by ERCP (endoscopic retrograde cholangiopancreatography) and surgery [4–9].

However, laparoscopic IOC and CBD exploration is technically demanding and time consuming and needs proper instrumentation for every single procedure: for all these reasons, it is far from being a commonplace in most surgical departments.

Current international guidelines provided by ASGE (American Society for Gastrointestinal Endoscopy) do not recommend routine IOC as a limited number of patients can benefit among all procedures performed [10]. Then, the accepted strategy is selective IOC based on the preoperative prediction model of risk [10, 11]. Selective IOC is nowadays performed in no more than 20% of the general surgery departments.

Therefore, the majority of the patients do not undergo intraoperative CBD evaluation during standard cholecystectomy and up to 5% of these patients presents retained ductal stones after surgery [12, 13].

We added routine partial cysticotomy with bile duct flushing during standard laparoscopic cholecystectomy in all patients with low risk of common duct stones.

## 2. Materials and Methods

Over a period of two years, between September 2013 and September 2015, 776 laparoscopic cholecystectomies (LC) have been performed for symptomatic GSD in the General Surgery Department of “Monaldi” Hospital in Naples: among them, 82 LC were performed during other laparoscopic procedures as, in main part, right colectomies and gastrectomies. Urgent procedures (45 cases) have been excluded for our analysis.

All 731 patients analyzed underwent preoperative clinical evaluation, liver functional tests, and abdominal ultrasound examination. Based on these features, patients were stratified into three categories of risk for carrying CBDS.

**Figure 1:**
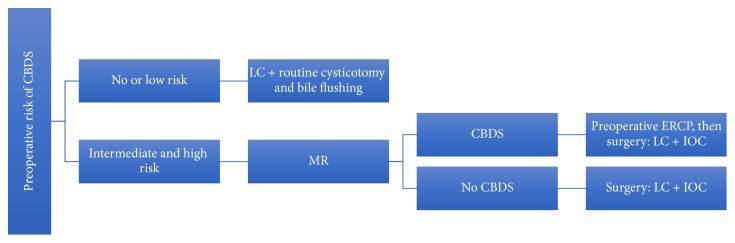
Flow-chart showing therapeutic strategy.

Population studied is summarized in Table 1 and treatment strategy in Figure 1.

Surgery was performed according to the classic priority criteria, after an average of time of 104 days (range 17–379 days), taking into account the first symptomatic event. Main reason for this delay of time was the availability of the operating room for a benign disease. An abdominal ultrasound evaluation was repeated close to the operation, when the preoperative radiologic workup was older than 30 days.

All patients underwent a modified LC operative technique with routine cysticotomy and main duct bile flushing.

Patients presenting with intermediate to high risk of CBDS according to the clinical algorithm, were referred to perform intraoperative cholangiography, although part of them have already undergone ERCP.

First postoperative visit was planned one week after surgery and then after two months.

All patients were clinically reevaluated in September 2016.

## 3. Modified LC Operative Technique

The patient is placed in supine position with legs abducted. Surgeon stands between the legs of the patient, first assistant/cameraman stands on the left side of the patient and second assistant on the right. Pneumoperitoneum is created performing an open approach into the umbilicus. We use to place trocar as in the North-American position, which permits an easier access to the CBD, facilitating transcystic introduction of the catheter for IOC, and a safer approach to the Calot's triangle.

**Figure 2:**
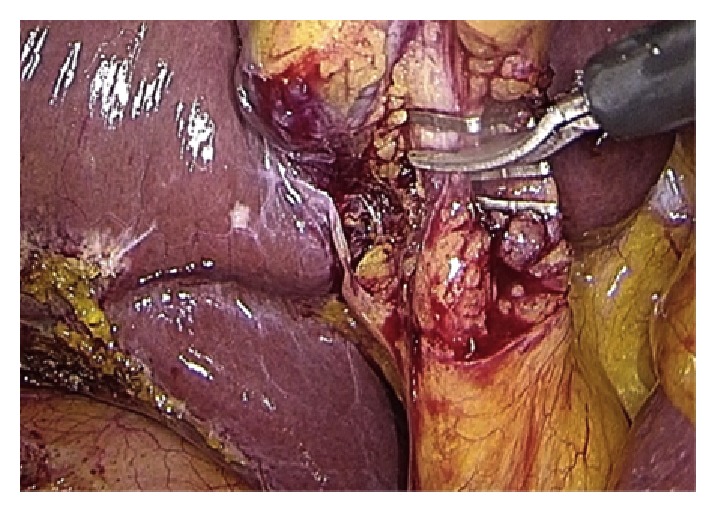
Partial cysticotomy.

**Figure 3:**
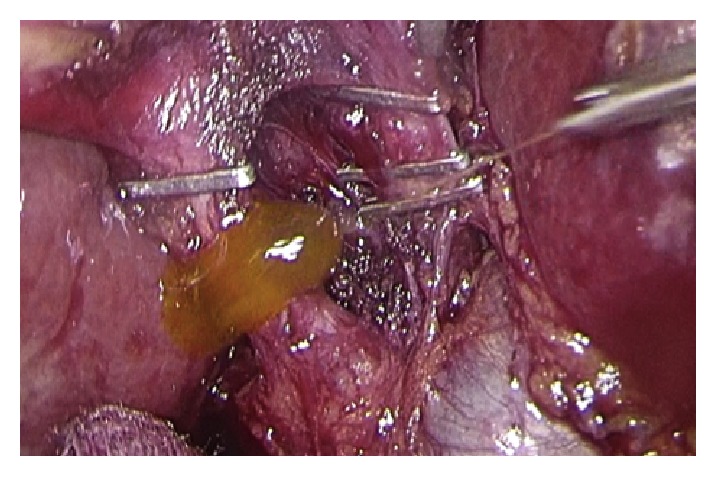
Flushing and bile outflow evaluation.

**Figure 4:**
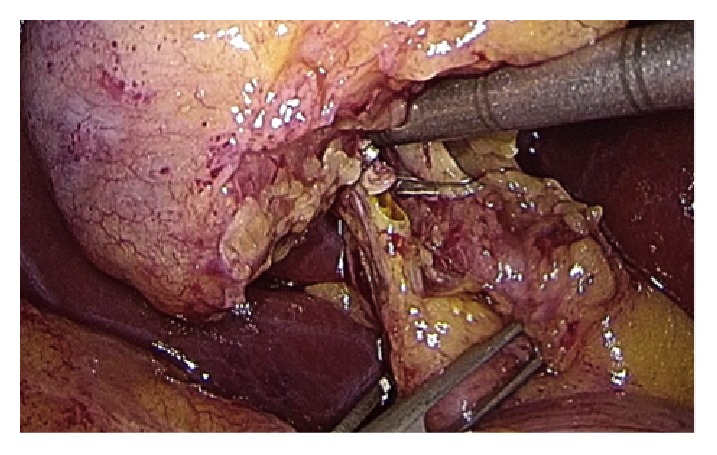
Gentle squeeze on the CBD and the cystic duct.

**Figure 5:**
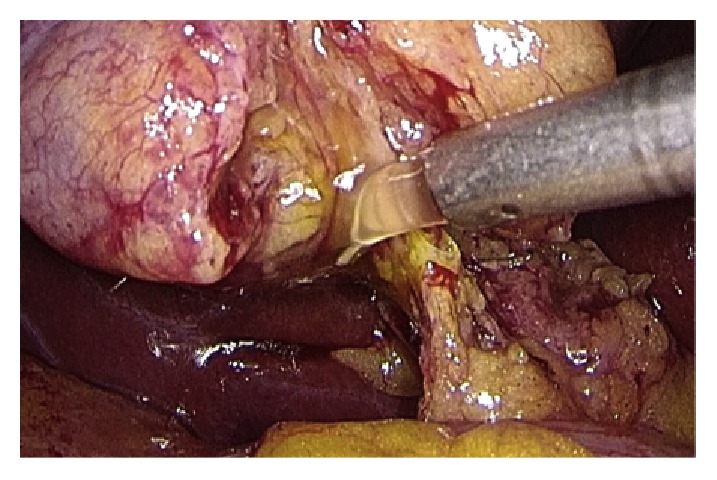
Bile rinsing.

**Figure 6:**
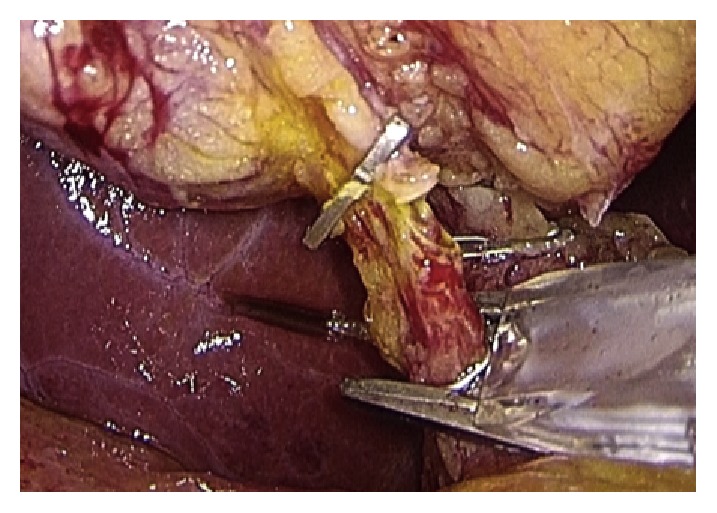
Clipping of the cystic duct.

**Figure 7:**
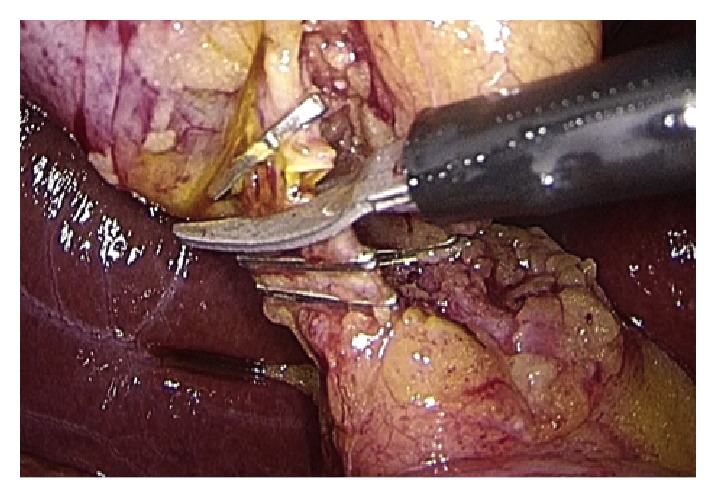
Cystic duct complete section.

Operative technique has largely been described. Performing a gentle dissection of fatty-areolar tissues of the Calot triangle, two structures running parallels into the gallbladder are exposed: cystic duct and cystic artery. Once cystic duct and artery are identified, a single 5 mm titanium clip is placed on the junction between the infundibulum and the cystic duct. Others three metallic clips are placed on the cystic artery, two proximal and one distal. A partial section of the cystic duct is performed, allowing bile to flow out (Figure 2). Bile thickness and tension of outflowing out are important features to evaluate (Figure 3). A gentle squeezing of the hepatic peduncle (Figure 4) is repeated while multiple washings are performed with an irrigator (Figure 5): action is repeated till bile becomes yellowy clear and fluid. When bile clearance is satisfactory, two metallic clips are placed to close proximally the cystic duct (Figure 6). Once the cystic duct is clipped and divided (Figure 7), the infundibulum is retracted cephalic and the gallbladder is mobilized from the liver. Gallbladder extraction is performed using a retrieval bag, subhepatic drainage is positioned, trocars removal is undertaken under vision, and trocar sites are sutured.

## 4. Results

Population studied accounts for 731 patients, undergone LC per GSD over 2 years. Male to female ratio was 1 : 1,7, the average age was 59 years (range 19–82), and the average BMI was 28,2 (range 20–39,5).

Patients were preoperatively stratified on the clinical risk of CBDS: 486 presented with no or low risk for CBDS, while 245 presented with intermediate to high one. Among these 245 patients, 127 had CBDS detected by the preoperative RM and were referred to a preoperative ERCP: 96 (75,6%) presented ductal stones, while 31 (24,4%) had no CBDS detected during the endoscopic procedure.

All these 127 patients underwent LC with IOC after ERCP (average time 4,6 days, range 3 to 14 days). In three cases (2.4%) over 127 round-shaped fill-defects were detected at cholangiography. The transcystic extraction of CBDS in these three cases was provided using Dormia basket.

**Figure 8:**
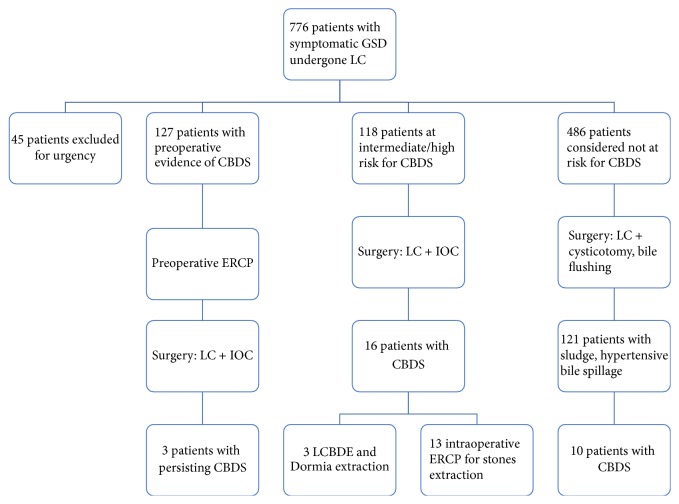
Flow-chart: population studied.

Patients with no evidence of CBDS at the preoperative MR but at risk for CBDS were candidate to LC with IOC. Among these 118 patients, 16 (13,5%) presented ductal fill-defects at IOC: 3, having smaller defects, were successfully managed using a Dormia basket, while 13 underwent intraoperative ERCP for stones extraction (see Figure 8).

Patients presenting with low preoperative risk of CBDS (486 cases) were candidate to LC with routine cysticotomy and bile flushing: 121 patients among them presented thick bile sludge, solid debrides, or augmented tension of the bile outflow while performing the maneuver. In all these 121 patients we proceed to IOC, which helped in detecting 10 cases (2%) of ductal stones: clearance of the CBD was achieved using a Dormia basket in this subgroup.

No intraoperative complications or conversion to open technique occurred.

Postoperative morbidity was 4% including medical conditions and umbilical trocar site infections (2,2%). Neither cholangitis nor pancreatitis occurred in the short postoperative period.

Average follow-up time was 22,8 months (range 12 to 37).

Nonspecific abdominal symptoms as dyspepsia, flatulence, and abdominal discomfort occurred in 114 patients (15,6%) after LC.

Two patients (0,3%) were diagnosed with retained ductal stones during this follow-up: both of them had been preoperatively considered at intermediate risk of CBDS.

## 5. Discussion

Laparoscopic cholecystectomy (LC) has become the gold standard for the treatment of GSD, since it was introduced in the latter eighties, for its proven safety and feasibility and for short-term advantages provided compared to the open technique [14, 15].

No univocal consensus, otherwise, has been reached yet on timing and management of CBDS in the era of LC [4, 5]. Certainly, preoperative workup lacks in sensitivity [11].

Unrecognized ductal stones can lead to undertreatment and potentially frightening conditions [1, 16–18]. Postoperative complications due to retained stones are described to be as high as 5%. GSD being an endemic disease, 5% accounts for a significant part of population.

Current international guidelines recommend selective IOC based on preoperative risk of CBDS. This attitude, actually, deals well with the frequent limited availability of both the equipment and the operating room. In any case, IOC is performed in no more than 20% of the general surgery departments.

We routinely employ the selective IOC strategy for GSD since the Nighties. However, we were found to have symptomatic residual ductal stones' rate as high as in literature.

Therefore, we modified LC's operative technique introducing a systematic noninvasive exploration of the CBD in order to help in integrating standard LC.

The manoeuvre is simple and fast. No injury of the main bile tract occurred in our experience. Taking 1.8 to maximum 4.2 minutes, it aims to realize the evaluation and the clearance of the bile into the CBD in a noninvasive manner.

Our criteria to further proceed to an unplanned IOC are presence of thick bile sludge, solid debrides, and/or augmented tension of the bile outflow.

As high as one in every four low-risk patients (121 patients over 486) presented one or more of these criteria and was referred to an unplanned IOC: in 10 cases (2%) unsuspected CBDS were detected and directly cleared.

Furthermore, advantages of the manoeuvre can be postulated as it could help in reducing pressure into the CBD in the immediate postoperative time.

We did not evidence any increase on postoperatory surgical site infection's rate. Umbilical trocar site infections accounted for 2,2% in our population. Neither postoperative cholangitis nor pancreatitis occurred in the short-term period.

After a median follow-up time of 22,8 months (range 12 to 37, no routine cholangiography is undertaken), the postoperatory rate of retained CBDS in our population was 0.3%.

## 6. Conclusions

Routine cysticotomy and bile flushing in our experience is a valid, simple, and not time consuming manoeuvre that can help in decompressing and flushing CBD, perhaps helping in reducing postoperative intraductal tension on the surgical clips. Furthermore, it is a valid tool for extending selective IOC approach in a focused manner.

Further evaluations have to be conducted to evaluate risks and effectiveness of this manoeuvre.

## Figures and Tables

**Table 1:** Population studied, stratified by preoperative risk of CBDS.

History of pancreatitis, cholangitis, jaundice	Altered LFTs or bilirubin level > 1,8 mg/dL	CBD > 6 mm	Probability of CBDS	*Patients (num)*	
−	−	−	Low	*486*	*486*
−	−	+	Intermediate	*83*	*131*
−	+	−	Intermediate	*11*	
+	−	−	Intermediate	*37*	
−	+	+	High	*23*	*114*
+	+	−	High	*42*	
+	−	+	High	*33*	
+	+	+	High	*16*	
